# Workplace mistreatment and problem drinking among physicians in Sweden: a longitudinal study

**DOI:** 10.5271/sjweh.4297

**Published:** 2026-09-01

**Authors:** Josefina Peláez Zuberbuhler, Alma Strömberg, Emelie Thern, Bodil J Landstad, Malin Sjöström, Emma Brulin

**Affiliations:** 1 Unit of Occupational Medicine, Institute of Environmental Medicine, Karolinska Institutet, Solna, Sweden.; 2 Department of Leadership and Organization, Kristiania University College, Oslo, Norway.; 3 Department of Health Sciences, Division of Public Health Science, Mid Sweden University, Östersund, Sweden.; 4 Unit of Research, Östersund Hospital, Östersund, Sweden.; 5 Department of Public Health and Clinical Medicine, Umeå University, Umeå, Sweden.; 6 Centre for Occupational and Environmental Medicine, Region Stockholm, Stockholm Sweden.

**Keywords:** healthcare, threat, violence, workplace mistreatment

## Abstract

**Objectives:**

Recent studies report high levels of workplace mistreatment among Swedish physicians. Various types of mistreatments have been correlated with adverse alcohol use. This study investigated the impact of workplace mistreatment (ie, threats and violence, harassment and incivility) on subsequent problem drinking among physicians in Sweden.

**Methods:**

Data from the Longitudinal Occupational Health survey in Healthcare Sweden (LOHHCS) were used, including 1618 clinically active physicians aged <67 years. Baseline data were collected between March and June 2022, and follow-up data between October and December 2023. Associations between three different types of workplace mistreatment and problem drinking were analyzed using logistic regressions adjusted for relevant confounders.

**Results:**

Exposure to threats and violence [odds ratio (OR) 2.78, 95% confidence interval (CI) 1.48–5.20] and workplace incivility (OR 3.25, 95% CI 1.24–8.54), respectively, significantly predicted problem drinking at follow-up. No significant association was found between harassment and problem drinking. When adjusting for co-occurring forms of mistreatment, only threats and violence remained a significant predictor.

**Conclusion:**

This study reveals a significant association between exposure to threats and violence at work and workplace incivility, respectively, and subsequent problem drinking among physicians. The findings underscore the need for interventions to prevent mistreatment within the healthcare system, as well as treatment and support for individuals who have been exposed.

Workplace mistreatment is widely recognized for its adverse physical and mental health outcomes, including adverse alcohol consumption, decreased efficiency, and lower job satisfaction (1). It encompasses a range of behaviors that deviate from accepted social norms, often marked by varying degrees of discriminatory undertones and a deliberate or perceived intent to inflict psychological or emotional harm on the recipient (2). Various forms of mistreatment at work exist; this study focuses on workplace incivility, identity-based harassment, and threats and violence (3, 4). They are prevalent across various professional settings, including the Swedish healthcare system, with perpetrators including patients, their relatives, and colleagues (3).

Workplace incivility involves low-intensity deviant behavior with ambiguous intent to harm, violating norms of mutual respect (5). In Sweden, every fourth physician reported experiencing workplace incivility in the past year from both patients and colleagues (3). Workplace incivility is known to have negative consequences, including affecting personal well-being and workplace performance (6), as well as impairing cognitive functions such as attention and memory, which can potentially compromise patient care (7). A review of 28 studies across eight countries found that workplace incivility has negative consequences for healthcare workers, including burnout, psychological distress, and reduced quality of care (8).

Identity-based harassment encompasses various forms of discriminatory behavior, including sexual, gender-based, and ethnic harassment. These behaviors involve unwelcome conduct often manifesting within power-imbalanced contexts and contributing to the creation of hostile work environments (9, 10). Exposure to these harassment types has been linked to mental health deterioration (11–13), impaired workplace communication (13), reduced self-confidence and job performance (12), increased sick leave (11), and negative career consequences, such as constraints on specialty choice (12). A recent Swedish study found that 27% of physicians experienced identity-based harassment in the past year, with women (36%) reporting considerably higher exposure than men (18%) and similarly elevated exposure among those with non-European backgrounds (39%) (3). Previous studies also indicate that female physicians are more frequently misidentified as non-physicians and face greater disrespect (14). In the US, non-Caucasian healthcare workers report higher rates of racial harassment and workplace discrimination compared to Caucasians (15).

Threats and violence within the Swedish healthcare system have increased significantly in recent years, with healthcare workers being more exposed to violence than other professions, including law enforcement (16). Workplace threats and violence have been associated with reduced life satisfaction, depression, suicidal ideation and increased malpractice risk, ultimately compromising patient care (4). An umbrella review of 32 studies found that the prevalence of workplace violence among healthcare workers was 14–36%, while threats were 30–43% (4). In Sweden, 12% of physicians reported experiencing threats and violence at work from patients or relatives in the past year, with higher rates among interns (18%) and those working in emergency (31%) and psychiatric care (35%) (17).

International longitudinal studies have correlated workplace mistreatment (eg, perceived sexual harassment and psychological humiliation) with subsequent problematic drinking habits. Specifically, Rospenda et al (18) found this association among men in a national sample in the US, while Richman et al (19) identified such correlations among both male and female physicians. More recently, gender-based harassment in the workplace has been identified as a significant etiological factor in alcohol-related morbidity and mortality, disproportionately affecting women (11). Given the indicated links between workplace mistreatment and problematic alcohol consumption, further investigation into potential risks across various forms of mistreatment (workplace incivility, identity-based harassment, threats and violence) is warranted among physicians (20). Alcohol consumption is a prevalent concern among physicians worldwide, and identifying the causes of problematic alcohol consumption is important. Reports identifying physicians as a high-risk group for alcohol-related issues are particularly concerning, given the potential implications for both their own well-being and patient safety (21, 22). The prevalence of problem drinking, ie, alcohol consumption that negatively affects an individual’s health, social relationships, and broader societal interactions (23), among Swedish physicians is 3.7% (21). The present study aims to examine the association between three forms of workplace mistreatment – workplace incivility, identity-based harassment and threats and violence – and subsequent levels of alcohol consumption (abstaining, moderate drinking, and problem drinking) among physicians in Sweden.

## Methods

Data were drawn from the 2022 and 2023 Longitudinal Occupational Health Survey in Healthcare Sweden (LOHHCS). A representative sample of 7908 physicians was taken from the Swedish occupational and educational registers at baseline in 2022. An invitation to participate was distributed in March 2022 with three reminders, and a total of 2712 (34.3%) responded. The LOHHCS cohort is an open cohort, meaning that at follow-up, those who had died, retired, migrated, or stopped working as physicians were excluded, and a new sample was drawn among the newly educated physicians. At follow-up, the survey was sent to 7780, of which 7294 were included in the 2022 survey cohort, and the response rate was 36.4%. For this study, we excluded those aged >67 years (retirement age). This resulted in a final analytical sample of 1618 physicians who responded to the questionnaire at both baseline and follow-up. Statistics Sweden conducted dropout and missing data analyses by comparing respondents to the overall sample and the target population using data from national registers (sex, ethnicity, age, and place of living and working, respectively). Their analysis revealed no evidence of systematic missing data. To assess potential loss to follow-up, individuals who responded at both baseline and follow-up (ie, the study sample) were compared with those who responded only at baseline, based on the variables sex, age, rank, workplace, hours worked per week, general health and burnout. This comparison also indicated no systematic differences between the groups, except that the dropouts had a higher share of burnout than those who responded both times.

The National Ethical Review Agency approved the study (Ref. No.: 2020-06613; 2021-05574-02; 2022-00310-02).

## Measures

### Outcome

*Alcohol consumption*. This construct was assessed using a question about alcohol consumption and a modified version of the Cut, Annoyed, Guilty, and Eye-opener (CAGE) questionnaire, originally developed by Ewing (24). Participants were first asked, “Do you regularly drink alcohol?” with response options presented on a 5-point Likert scale ranging from 1 (never) to 5 (every day). Individuals who responded 2–5 proceeded to complete the CAGE questionnaire, which comprises four items evaluating their lifetime drinking experience (eg, “Have you ever felt you should cut down on your drinking?”). Each item was scored “no” or “yes.” Consistent with Ewing’s validation study (24), a score of ≥2 affirmative responses indicated clinically significant problem drinking. Because the CAGE questionnaire is only administered to individuals who report alcohol use, we additionally included abstainers as a separate category. Thus, a nominal outcome variable with three categories was created: abstaining (ie, those answering “never” to the first question), moderate drinking (ie, scores <2 on the CAGE scale), and problem drinking (ie, scores ≥2 on the CAGE scale).

### Exposures

All items referred to mistreatment experienced at work, regardless of whether the perpetrator was a colleague, patient, or relative.

*Workplace incivility* was assessed using Schad et al’s Swedish-translated version (25) of Cortina et al’s Workplace Incivility Scale (26). The scale comprises seven items, each rated on a 5-point Likert scale ranging from 0 (never/almost never) to 4 (every day). Participants were asked to indicate whether, in the past 12 months, they had experienced behaviors from others at work such as being “put down or treated condescendingly” or “ignored or excluded from professional camaraderie”. The scale demonstrated high internal consistency, with a Cronbach’s alpha of 0.88. Following Cortina et al’s recommendations (26), respondents were categorized into two groups: (i) no/low exposure (0–11 points) and (ii) moderate/high exposure (11–28 points). In the present study, workplace incivility exposure was operationalized as moderate-to-high exposure.

*Identity-based harassment* was evaluated by asking participants how frequently they had encountered specific forms of harassment at work in the past 12 months (3). This included three items: (i) exposure to unwelcome advances or offensive remarks of a sexual nature, (ii) experiences of derogatory or condescending treatment related to gender, and (iii) exposure to harassment or discrimination based on ethnicity or skin color. Responses were recorded on a 4-point Likert scale, ranging from 1 (not exposed in the last 12 months) to 4 (≥1 times a week). Exposure to identity-based harassment was assigned to participants who reported ≥1 occurrence, ie, experiencing it ≥1 times a week to ≥1 times in the last 12 months, across any of the three harassment items.

*Threats and violence* at the workplace were assessed with the question: “In the past 12 months, have you been exposed to violence or threats of violence at work?” Responses were recorded on a 4-point Likert scale ranging from 1 (never) to 4 (≥1 times per week). For analysis, responses were dichotomized into “yes” (exposed) and “no” (not exposed), with “yes” including all responses except “never.”

### Demographics

Demographic data, including sex (male or female), year of birth, and country of birth (Sweden, inside Europe, or outside Europe), were obtained from the Swedish Longitudinal Integrated Database for Health Insurance and Labour Market Studies (LISA). Additional demographic variables were collected through survey responses, including professional rank (categorized as junior/intern, resident, specialist, or consultant) and workplace setting (primary care, hospital, or other).

### Data analysis

Descriptive statistics were used to examine the prevalence of abstaining, drinking, and problem drinking (follow-up) across the study variables (baseline). Descriptives also show the new cases of abstaining and problem drinking, respectively, as well as the share of new cases across exposure confounders.

Next, to assess the probability of reporting abstaining, drinking, and problem drinking at follow-up in relation to baseline workplace mistreatment, multinomial logistic regression analyses were conducted. Moderate drinking was set as the base category, meaning that the analysis will identify the probability (or odds) of reporting abstaining and problem drinking relative to the odds of reporting moderate drinking when exposed to workplace mistreatment. Three separate models were tested. The first model (univariate model 1) included each type of workplace mistreatment separately. The second model (model 2) examined mistreatment types separately (models 2a, 2b, and 2c, respectively) while adjusting for baseline alcohol consumption and confounders. In the third model (model 3), all three mistreatment variables were included jointly, adjusting for baseline alcohol consumption and confounders. Baseline alcohol consumption was included as a three-category variable (abstaining, moderate drinking, and problem drinking). Confounders included sex, professional rank, workplace setting, and country of birth. Age was not included in the adjusted models due to its strong correlation with professional rank, in order to avoid multicollinearity. The results were reported as odds ratios (OR) with corresponding confidence intervals (CI) and P-values, with statistical significance set at P≤0.05. Finally, to test the robustness of the results, sensitivity analyses were conducted, in which baseline cases of problem drinking and abstaining were excluded, respectively. Given the previously identified sex differences, we also performed a sex-stratified sensitivity analysis.

All statistical analyses were performed using IBM Statistical Package for Social Sciences (SPSS) software, version 29.0. To enhance the robustness of the analyses, all models were adjusted for strata based on healthcare administrative regions (N=23).

## Results

### Descriptive statistics

Table 1 shows the descriptive statistics for the respective categories of alcohol consumption, ie, abstaining, moderate drinking, and problem drinking at follow-up. The table also reports the number of participants who exhibited abstaining or problem drinking at follow-up who were not meeting these conditions at baseline. The study sample comprised 1618 physicians, of whom 58.6% (N=948) were female, and the mean age in 2022 was 43.8 (SD=10.9) years. In terms of professional rank, specialists constituted the largest subgroup (36.0%), whereas junior and intern physicians represented the smallest proportion (10.7%). Most participants were employed in hospital settings (56%), and being born in Sweden was overrepresented (83.3%). Regarding workplace mistreatment, the prevalence of identity-based harassment was the highest (27.1%), followed by threats and violence at 22.3% and workplace incivility at 6.3%.

**Table 1:** Descriptive statistics for abstaining, moderate drinking, and problem drinking.

		Total		Abstaining^c^		Moderate drinking^c^		Problem drinking^c^		New abstainers^d^		New problem drinkers^e^
		N (%)		N (%)		N (%)		N (%)		N (%)		N (%)
Total		1618		236 (14.6)		1310 (81.1)		69 (4.3)		73 (5.3)		38 (2.4)
Gender												
	Male	670 (41.4)		95 (40.3)		538 (41.1)		34 (49.3)		28 (38.4)		21 (55.3)
	Female	948 (58.6)		141 (59.7)		772 (58.9)		35 (50.7)		45 (61.6)		17 (44.7)
Rank												
	Junior and intern physician	172 (10.7)		28 (11.9)		133 (10.2)		10 (14.5)		8 (11.1)		8 (21.1)
	Resident	443 (27.6)		65 (27.7)		366 (28.2)		11 (15.9)		19 (26.4)		9 (23.7)
	Specialist	578 (36.0)		92 (39.1)		461 (35.5)		25 (36.2)		23 (31.9)		13 (34.2)
	Consultant	413 (25.7)		50 (21.3)		339 (26.1)		23 (33.3)		22 (30.6)		8 (21.1)
Worksite												
	Primary care	558 (34.5)		98 (41.5)		440 (33.6)		18 (26.1)		26 (35.6)		10 (26.3)
	Hospital	905 (56.0)		114 (48.3)		744 (56.8)		47 (68.1)		39 (53.4)		24 (63.2)
	Other	154 (9.5)		24 (10.2)		125 (9.5)		4 (5.8)		8 (11)		4 (10.5)
Country of birth												
	Sweden	1338 (83.2)		161 (68.8)		1108 (85.1)		67 (97.1)		52 (72.2)		36 (94.7)
	Inside Europe ^a^	201 (12.5)		42 (17.9)		159 (12.2)		0 (0)		15 (20.8)		0 (0)
	Outside Europe	69 (4.3)		31 (13.2)		35 (2.7)		2 (2.9)		5 (6.9)		2 (5.3)
Workplace incivility ^b^		102 (6.3)		27 (11.6)		69 (5.3)		6 (8.7)		10 (13.7)		5 (13.2)
Identity harassment		436 (27.1)		78 (33.2)		338 (25.9)		19 (27.5)		24 (33.3)		11 (28.9)
Threats and violence		361 (22.3)		60 (25.4)		277 (21.2)		23 (33.3)		17 (23.3)		15 (39.5)

^a^ Within Europe except Sweden. ^b^ Moderate/high exposure to workplace incivility. ^c^ Participants at follow up. ^d^ Participants reporting abstaining at follow-up who were not abstainers at baseline. ^e^ Participants reporting problem drinking at follow-up who were not problem drinkers at baseline.

With respect to alcohol consumption at follow-up, nearly 15% of physicians reported abstaining from alcohol in the past 12 months, whereas 4.3% met the criteria for problem drinking, with slight variations observed across demographic groups. The proportion of men was somewhat higher among physicians with problem drinking (49.3%) than among abstainers (40.3%) and moderate drinkers (41.1%), although the sex distribution was relatively similar overall. Differences across professional rank and workplace setting were modest, although specialists and hospital-based physicians constituted the largest share of individuals with problem drinking. Finally, among the different forms of workplace mistreatment, problem drinking was most prevalent among those exposed to threats and violence.

At follow-up, 2.4% were new cases of problem drinkers, of which most were men (55.3%), specialist physicians (34.2%), and working in a hospital setting (63.2%). Among the new cases of problem drinking, 13.2% reported workplace incivility at baseline. The respective figures for identity-based harassment were 28.9% and threats and violence were 39.5%. We also identified 73 new cases of abstainers, among which 13.7% reported baseline workplace incivility, 33.3% identity-based harassment, and 23.3% threats and violence.

### Impact of workplace mistreatment on alcohol consumption

The association between workplace mistreatment and subsequent alcohol consumption is presented in table 2. Starting with abstaining, results show (model 2a) that physicians who experienced workplace incivility at baseline had approximately twofold higher odds of reporting abstaining versus moderate drinking at follow-up, after adjusting for confounders and baseline alcohol consumption (OR 2.05, 95% CI 1.07–3.91). This association remained of similar magnitude in model 3, when additionally adjusting for co-occurring forms of mistreatment. In contrast, the association between identity-based harassment and abstaining attenuated after adjustment, suggesting potential confounding or mediation by other factors (model 2b). No clear association was observed between threats and violence and abstaining.

**Table 2:** Multinomial logistic regression for the association between workplace mistreatment and follow-up alcohol consumption, with moderate drinking as the base-category. [OR=odds ratio; CI=confidence interval; NA=redundant].

		Model 1^a^			Model 2a^b^			Model 2b^c^			Model 2c^d^			Model 3^e^	
		OR (95% CI)	P-value		OR (95% CI)	P-value		OR (95% CI)	P-value		OR (95% CI)	P-value		OR (95% CI)	P-value
**Abstaining**															
Workplace incivility		1.56 (1.15–2.11)	0.004		2.05 (1.07–3.91)	0.030								2.03 (1.03–4.00)	0.039
Identity-based harassment		1.42 (1.05–1.91)	0.021					1.19 (0.78–1.82)	0.422					1.09 (0.69–1.73)	0.690
Threats & violence		1.27 (0.92–1.75)	0.147								1.01 (0.66–1.57)	0.945		0.92 (0.58–1.46)	0.716
Male (ref female)					0.98 (0.67–1.43)	0.903		1.01 (0.68–1.49)	0.952		0.99 (0.68–1.46)	0.998		0.92 (0.57–1.46)	0.886
Rank															
	Junior & intern physician				1.41 (0.72–2.75)	0.315		1.33 (0.67–2.63)	0.416		1.41 (0.72–2.76)	0.320		1.38 (0.69–2.73)	0.361
	Resident				0.86 (0.49–1.51)	0.608		0.86 (0.49–1.52)	0.606		0.90 (0.52–1.58)	0.724		0.84 (0.48–1.49)	0.562
	Specialist				0.87 (0.49–1.56)	0.605		0.86 (0.48–1.53)	0.606		0.89 (0.50–1.58)	0.684		0.85 (0.48–1.52)	0.593
	Consultant				.	.		.	.		.	.		.	.
Workplace															
	Primary care				1.26 (0.62–2.56)	0.516		1.19 (0.59–2.41)	0.625		1.21 (0.60–2.43)	0.598		1.23 (0.61–2.48)	0.569
	Hospital				0.96 (0.49–1.86)	0.901		0.95 (0.49–1.84)	0.876		0.96 (0.49–1.85)	0.894		0.95 (0.49–1.84)	0.870
	Other														
Country of birth															
	Sweden				0.31 (0.14–0.66)	0.002		0.29 (0.14–0.62)	0.001		0.27 (0.13–0.58)	<0.001		0.31 (0.14–0.68)	0.003
	Inside Europe^f^				0.42 (0.18–1.01)	0.052		0.38 (0.16–0.90)	0.028		0.38 (0.16–0.91)	0.029		0.41 (0.17–0.97)	0.043
	Outside Europe				.	.		.	.		.	.		.	.
Baseline alcohol consumption^g^					0.03 (0.02–0.04)	<.001		0.027 (0.02–0.04)	<.001		0.03 (0.02–0.04)	<.001		0.03 (0.02–0.04)	<0.001
**Problem drinking**															
Workplace incivility		1.40 (0.83–2.38)	0.211		3.25 (1.24–8.54)	0.016								2.36 (0.85–6.51)	0.098
Identity-based Harassment		1.08 (0.63–1.87)	0.776					1.49 (0.76–2.93)	0.250					1.00 (0.47–2.10)	1.000
Threats & violence		1.86 (1.11–3.12)	0.019								2.78 (1.48–5.20)	0.001		2.65 (1.36–5.18)	0.004
Male (ref female)					1.55 (0.87–2.75)	0.135		1.59 (0.89–2.87)	0.119		1.52 (0.86–2.69)	0.152		1.68 (0.92–3.06)	0.091
Rank															
	Junior & intern physician				1.168 (0.474–2.881)	0.736		1.17 (0.47–2.92)	0.740		1.06 (0.43–2.62)	0.897		1.00 (0.39–2.54)	0.995
	Resident				0.507 (0.214–1.198)	0.121		0.56 (0.24–1.33)	0.189		0.50 (0.21–1.17)	0.111		0.48 (0.20–1.17)	0.109
	Specialist				1.028 (0.455–2.323)	0.948		1.06 (0.47–2.39)	0.893		1.05 (0.46–2.39)	0.903		1.01 (0.44–2.31)	1.009
	Consultant				.	.		.	.		.	.		.	.
Workplace															
	Primary care				0.91 (0.26–3.11)	0.516		0.91 (0.26–3.19)	0.886		1.07 (0.30–3.86)	0.917		0.94 (0.27–3.24)	0.925
	Hospital				1.36 (0.44–4.22)	0.901		1.49 (0.47–4.73)	0.500		1.75 (0.53–5.74)	0.357		1.43 (0.46–4.46)	0.533
	Other				.	.		.	.		.	.		.	.
Country of birth															
	Sweden				0.58 (0.13–2.57)	0.473		0.56 (0.13–2.46)	0.443		0.49 (0.11–2.16)	0.343		0.05 (0.12–2.35)	0.399
	Inside Europe ^f^				NA	NA		NA	NA		NA	NA		NA	NA
	Outside Europe				.	.		.	.		.	.		.	.
Baseline alcohol consumption ^g^					48.85 (24.66–96.79)	<.001		43.90 (22.42–85.96)	<0.001		45.71 (23.16–90.23)	<0.001		57.33 (27.99–117.43)	<0.001

^a^ Univariate model ^b^ Model 2a: -2 log likelihood=383.531 (P<0.001). ^c^ Model 2b: -2 log likelihood=435.762 (P<0.001). ^d^ Model 2c. -2 log likelihood=442.291(P<0.001). ^e^ Model 3. -2 log likelihood=661.949 (P<0.001). ^f^ Within Europe except Sweden. ^g^ Baseline alcohol consumption was entered as a categorical covariate with three levels: abstaining, moderate drinking, and problem drinking.

Turning to problem drinking (model 2a), exposure to workplace incivility at baseline was associated with more than threefold higher odds of reporting problem drinking at follow-up compared to those who were classified as moderate drinkers when adjusting for baseline alcohol consumption (OR 3.25, 95% CI 1.24–8.54). Similarly, as shown in model 2c, physicians exposed to threats and violence had nearly threefold higher odds of problem drinking at follow-up (OR 2.78, 95% CI 1.48–5.20), suggesting a substantial increase in risk.

When all three types of mistreatments were included simultaneously in the model (model 3), the magnitude of the association between workplace incivility and problem drinking remained elevated (OR 2.36), although the estimate was less precise (P=0.098). In contrast, the association between threats and violence and problem drinking remained robust, with more than twofold increase in odds (OR 2.65, 95% CI 1.36–5.18).

### Sensitivity analysis

Additional sensitivity analyses were conducted (supplementary material, www.sjweh.fi/article/4297, tables S1 and S2), confirming the results presented above. In the first set of analyses, we excluded all individuals who reported abstaining at baseline (table S1). The overall pattern and magnitude of associations remained similar to the main analysis. In the second set, we excluded all physicians who reported problem drinking at baseline (table S2). Again, the findings were consistent with those of the main analysis, with only minor variations in effect estimates. In analyses stratified by sex (table S3), the association between exposure to threats and violence and subsequent problem drinking was more pronounced and statistically significant among women (OR 3.30, 95% CI 1.25–8.76), whereas estimates for men were in the same direction but not statistically significant (OR 2.13, 95% CI 0.80–5.65). Workplace incivility and identity-based harassment were not significantly associated with problem drinking. We found no statistically significant results related to abstaining.

## Discussion

This one-year follow-up panel study contributes important insights into the association between different forms of workplace mistreatment (workplace incivility, identity-based harassment, and threat and violence) and subsequent alcohol consumption among physicians in Sweden. Findings revealed that 22.3% of physicians reported exposure to threats and violence at work, a figure significantly higher than the 12% previously reported by the Swedish Medical Association in 2022 (17). Notably, such exposure was associated with an increased likelihood of reporting subsequent problem drinking. Further, 6.3% reported being exposed to workplace incivility, which was associated with increased odds of both abstaining and problem drinking at follow-up. In addition, 27% of participating physicians reported experiencing identity-based harassment, which did not appear to be associated with alcohol consumption. These results underscore the prevalence and impact of workplace mistreatment in Swedish healthcare settings, further corroborating earlier findings on the adverse consequences of mistreatment on healthcare workers (1, 3, 8, 27).

Research shows that threats and violence against healthcare workers in general, and physicians in particular, have increased over the last few decades (28). Meanwhile, many violent or threatening situations are never reported (28, 29). While previous studies have linked threats and violence to adverse outcomes for healthcare providers and patients, including impaired mental health and diminished quality of care (21, 30, 31), methodological limitations hamper any inferential investigations of these previous findings (28, 32). By employing a longitudinal panel design, our study strengthens the evidence base by suggesting a temporal relationship between exposure to workplace threats and violence and subsequent problem drinking among physicians, with more than a twofold increase in odds observed. These findings highlight the urgent need to address threats and violence in Swedish healthcare settings. However, a review concludes that few evidence-based organizational, legal, and regulatory interventions exist to stem the increasing prevalence of threats and violence in healthcare (28), indicating an important gap in knowledge.

Workplace incivility demonstrated a complex relationship to alcohol consumption, as it was linked to both abstaining and problem drinking at follow-up. These findings suggest that workplace incivility may influence drinking behavior in divergent ways, potentially reflecting different coping responses or avoidance behaviors among affected individuals. Another plausible explanation is that the observed association between incivility and problem drinking may be explained by co-occurring exposure to other forms of mistreatment (3) or that more overt and potentially traumatic experiences of incivility exert a stronger influence on maladaptive coping behaviors such as problem drinking. Additionally, previous studies have demonstrated that perpetrators of workplace incivility are more often colleagues than patients (3). In contrast, harassment (3) as well as threats and violence are primarily directed at physicians by patients or their relatives (17, 29), which may influence coping strategies. Nevertheless, the fact that workplace incivility has been linked to adverse patient outcomes (33), turnover intention (34), mental ill health (32), and even suicide ideation (31) indicates that employers need to take it seriously and that further longitudinal research is needed to explore the negative effects of workplace incivility.

Additional sex stratified analysis indicated a stronger association between threats and violence and problem drinking among women than men, both aligning (20) and disagreeing with previous research (19). While the result must be interpreted with caution, the pattern may reflect variation in exposure between men and women (3). These findings highlight the potential importance of considering gender-specific mechanisms when examining the consequences of workplace mistreatment. Addressing mistreatment in the healthcare workplace should be considered a priority, not only to safeguard staff but also to prevent downstream effects on physicians’ health and well-being and, ultimately, on patient safety. According to Swedish labor regulations, the employer is obliged to organize work to prevent the risk of violence or threat and other forms of mistreatment as far as possible and to have specific safety procedures in place for work that may involve a risk of violence or threat. Meanwhile, threats and violence against healthcare workers are normalized as part of patient diagnoses and viewed as part of the work (35). Thus, institutional policies and interventions aimed at deformalizing, managing, and preventing workplace mistreatment, particularly threats and violence, are essential.

This study focuses on the associations between workplace mistreatment and subsequent alcohol consumption and not the reverse relationship. McFarlin et al (36) found that heavy drinking was associated with both victimization and perpetration of verbal and physical aggression at work, suggesting that reciprocal effects may also exist. This should be further researched using multiple measuring points. Additionally, considering the stigma associated with alcohol consumption and mental health challenges among physicians (37), awareness and support programs could help in identifying and supporting individuals at risk, thereby fostering healthier coping strategies.

### Strengths and limitations

The strengths of this study include its large, nationally representative sample of physicians in Sweden and the longitudinal design, which enhances the ability to explore temporal relationships between workplace mistreatment and problem drinking. By examining multiple forms of mistreatment and adjusting for baseline alcohol consumption and co-occurring exposures, the analyses provide a more nuanced and isolated understanding of each exposure’s impact on problem drinking.

However, several limitations must be acknowledged. The reliance on self-reported data introduces the risk of response bias, such as underreporting due to social desirability or fear of stigma (38). Recall bias may also affect accuracy, given that participants were asked to report experiences over the past year. Furthermore, the study focuses on a specific occupational group—physicians in Sweden—potentially limiting the generalizability of the findings to other healthcare workers or international contexts. Additionally, only individuals who responded to both waves of the survey were included, which may have introduced selection bias and reduced the overall sample size. While the total sample was relatively large, the sizes of certain subgroups—particularly those reporting workplace incivility and problem drinking—were limited, constraining the ability to draw robust generalizations for these specific groups.

The use of a validated clinical cutoff for the CAGE measure may have reduced variability due to dichotomization. However, supplementary analyses using the full CAGE score yielded similar results. In addition, although all exposure measures referred to mistreatment experienced at work, we did not differentiate between types of perpetrators (eg, colleagues, patients, or relatives) in the analysis, due to overlap and missing data. From a previous study (3), we know that perpetrators vary by profession, ethnicity and gender, and across types of mistreatment, with incivility more often involving colleagues and identity-based harassment more often involving patients or relatives. These differences may be important for understanding coping responses and for designing targeted interventions. Future studies should therefore investigate the role of perpetrator type in relation to alcohol-related outcomes. Different sources of mistreatment (eg, patients versus colleagues) may require different types of preventive strategies, which should be explored in future research.

### Concluding remarks

The findings of this study demonstrate a significant association between exposure to workplace threats and violence and subsequent problem drinking among physicians. These results underscore the urgent need for preventive interventions targeting threats and violence within the healthcare environment, as well as the development of appropriate treatment and support systems for affected individuals. Future research is also of value in exploring underlying mechanisms and potential protective factors further.

## Supplementary material

Supplementary material
