# Supplementary material for: Workplace mistreatment and problem drinking among physicians in Sweden: a longitudinal study
Source: Scand J Work Environ Health. 2026 Aug 31;52(5):540–8. doi: 10.5271/sjweh.4297 (PMC13550692; doi:10.5271/sjweh.4297)
Supplement: Supplementary file 1 [file SJWEH-52-540-S001.pdf]

Workplace mistreatment and problem drinking among physicians in Sweden: a longitudinal study[1]

by Josefina Peláez Zuberbuhler [2], Alma Strömberg, Emelie Thern, Bodil J Landstad, Malin Sjöström, Emma Brulin

1. Supplementary material
2. Correspondence to: Josefina Peláez Zuberbuhler, Unit of Occupational Medicine, Institute of Environmental Medicine, Karolinska Institutet, Solna, Sweden. [E-mail: josefina.pelaez.zuberbhler@ki.se]

Table S1. Multinomial logistic regression for the association between workplace mistreatment and follow-up alcohol consumption, with moderate drinking as the base-category, excluding abstainers at baseline

	Model 1[a]		Model 2a[b]		Model 2b[c]		Model 2c[d]		Model 3[e]	
	**OR (95% CI)**	**P-value**	**OR (95% CI)**	**P-value**	**OR (95% CI)**	**P-value**	**OR (95% CI)**	**P-value**	**OR (95% CI)**	**P-value**
**Abstaining**										
Workplace incivility	2.90 (1.42-5.92)	0.003	2.32 (1.08-4.99)	0.031					2.20 (0.99-4.90)	0.054
Identity-based harassment	1.45 (0.88-2.41)	0.147			0.73 (0.42-1.26)	0.257			1.22 (0.68-2.18)	0.499
Threats and violence	1.15 (0.66-2.02)	0.615					0.94 (0.52-1.69)	0.844	0.95 (0.52-1.75)	0.873
Male (ref female)			0.77 (0.47-1.29)	0.326	0.79 (0.47-1.34)	0.387	0.78 (0.47-1.28)	0.327	0.77 (0.46-1.30)	0.336
Rank										
Junior and intern physician			1.03 (0.43-2.45)	0.943	0.98 (0.41-2.34)	0.956	1.07 (0.45-2.55)	0.882	0.97 (0.40-2.34)	0.947
Resident			0.77 (0.39-1.53)	0.459	0.75 (0.37-1.52)	0.431	0.83 (0.42-1.65)	0.591	0.74 (0.36-1.49)	0.392
Specialist			0.67 (0.32-1.38)	0.277	0.65 (0.31-1.34)	0.242	0.68 (0.33-1.41)	0.303	0.64 (0.31-1.33)	0.228
Consultant			.	.	.	.	.	.	.	.
Workplace										
Primary Care			1.28 (0.51-3.20)	0.599	1.17 (0.47-2.93)	0.736	1.21 (0.48-3.02)	0.684	1.22 (0.48-3.08)	0.672
Hospital			0.90 (0.38-2.10)	0.806	0.87 (0.37-2.03)	0.748	0.89 (0.38-2.10)	0.785	0.88 (0.38-2.07)	0.774
Other			.	.	.	.		.	.	.
Country of birth										
Sweden			0.29 (0.11-0.80)	0.017	0.29 (0.11-0.80)	0.017	0.26 (0.09-0.71)	0.009	0.30 (0.11-0.84)	0.022
Inside Europe[f]			0.62 (021-1.87)	0.399	0.60 (0.20-1.82)	0.366	0.59 (0.19-1.79)	0.354	0.59 (0.19-1.80)	0.350
Outside Europe			.	.	.	.	.	.	.	.

Baseline alcohol consumption[g]			1.07 (0.14-8.22)	0.945	1.02 (0.13-7.75)	0.988	0.95 (0.12-7.27)	0.965	1.15 (1.50-8.86)	0.891
**Problem Drinking**										
Workplace incivility	1.74 (0.73-4.17)	0.214	3.28 (1.25-8.60)	0.016					2.37 (0.86-6.54)	0.096
Identity-based harassment	1.10 (0.64-1.90)	0.718			0.66 (0.34-1.31)	0.239			1.01 (0.48-2.12)	0.983
Threats and violence	1.90 (1.13-3.19)	0.015					2.78 (1.48-5.21)	0.001	2.65 (1.36-5.19)	0.004
Male (ref female)			1.53 (0.86-2.72)	0.146	1.58 (0.88-2.84)	0.128	1.50 (8.48-2.66)	0.163	1.66 (0.91-3.03)	0.098
Rank										
Junior and intern physician			1.15 (0.47-2.83)	0.764	1.15 (0.46-2.87)	0.765	1.05 (0.42-2.59)	0.921	0.98 (0.39-2.49)	0.972
Resident			0.50 (0.21-1.19)	0.119	0.56 (0.23-1.32)	0.184	0.49 (0.21-1.17)	0.109	0.48 (0.20-1.16)	0.105
Specialist			1.01 (0.45-2.29)	0.972	1.04 (0.46-2.36)	0.917	1.04 (0.46-2.36)	0.925	0.99 (0.43-2.28)	0.990
Consultant			.	.	.	.	.	.	.	.
Workplace										
Primary Care			0.91 (0.27-3.11)	0.880	0.91 (0.26-3.19)	0.886	1.07 (2.97-3.86)	0.916	0.94 (0.27-3.24)	0.926
Hospital			1.35 (0.44-4.21)	0.600	1.48 (0.46-4.71)	0.506	1.74 (0.53-5.72)	0.361	1.43 (0.46-4.45)	0.538
Other			.	.	.	.		.	.	.
Country of birth										
Sweden			0.58 (0.13-2.58)	0.477	0.56 (0.13-2.50)	0.453	0.49 (0.11-2.18)	0.351	0.53 (0.12-2.37)	0.403
Inside Europe[f]			.	.					.	.
Outside Europe			.	.	.	.	.	.	.	.
Baseline alcohol consumption[g]			51.19 (25.62-102.29)	<0.001	45.91 (23.25-90.66)	<0.001	47.68 (23.97-94.83)	<0.001	60.31 (29.18-124.65)	<0.001

[a] Univariate mode
[b] Model 2a: -2 log likelihood=263.460 (P<0.001).
[c] Model 2b: -2 log likelihood=301.645(P<0.001).
[d] Model 2c. -2 log likelihood=302.637 (P<0.001).
[e] Model 3. -2 log likelihood=415.929 (P<0.001).
[f] Within Europe except Sweden;
[g] Baseline alcohol consumption was entered as a categorical covariate with three levels: abstaining, moderate drinking, and problem drinking.

Table S2. Multinomial logistic regression for the association between workplace mistreatment and follow-up alcohol consumption, with moderate drinking as the base-category, excluding problem drinkers at baseline

	Model 1[a]		Model 2a[b]		Model 2b[c]		Model 2c[d]		Model 3[e]	
	**OR (95% CI)**	**P-value**	**OR (95% CI)**	**P-value**	**OR (95% CI)**	**P-value**	**OR (95% CI)**	**P-value**	**OR (95% CI)**	**P-value**
**Abstaining**										
Workplace incivility	2.34 (1.46-3.74)	<.001	2.08 (1.08-3.99)	0.027					2.12 (1.07-4.19)	0.030
Identity-based harassment	1.36 (1.01-1.84)	0.043			1.15 (0.75-1.76)	0.532			1.06 (0.67-1.69)	0.792
Threats and violence	1.23 (0.89-1.70)	0.203					0.97 (0.63-1.51)	0.894	0.87 (0.55-1.41)	0.587
Male (ref female)			0.95 (0.65-1.41)	0.826	0.98 (0.66-1.46)	0.941	0.98 (0.67-1.44)	0.923	0.94 (0.63-1.41)	0.780
Rank										
Junior and intern physician			1.43 (0.73-2.80)	0.300	1.36 (0.68-2.70)	0.384	1.44 (0.73-2.83)	0.297	1.42 (0.71-2.83)	0.323
Resident			0.83 (0.47-1.47)	0.529	0.84 (0.47-1.48)	0.540	0.87 (0.49-1.54)	0.640	0.82 (0.46-1.46)	0.499
Specialist			0.86 (0.48-1.54)	0.616	0.84 (0.47-1.52)	0.577	0.87 (0.49-1.56)	0.649	0.84 (0.47-1.51)	0.567
Consultant			.	.	.	.	.	.	.	.
Workplace										
Primary Care			1.27 (0.63-2.58)	0.504	1.19 (0.59-2.43)	0.618	1.21 (0.59-2.46)	0.597	1.24 (0.61-2.52)	0.556
Hospital			0.94 (0.48-1.84)	0.863	0.93 (0.47-1.82)	0.834	0.93 (0.48-1.82)	0.839	0.93 (0.47-1.82)	0.831
Other			.	.	.	.	.	.	.	.
Country of birth										
Sweden			0.42 (0.17-1.00)	0.051	0.37 (0.16-0.89)	0.027	0.38 (0.16-0.91)	0.030	0.31 (0.14-0.67)	0.003
Inside Europe[f]			0.30 (0.14-0.65)	0.002	0.29 (0.13-0.61)	0.001	0.27 (0.13-0.58)	<.001	0.40 (0.16-0.97)	0.042
Outside Europe			.	.	.	.	.	.	.	.
Baseline Alcohol consumption[g]			0.03 (0.02-0.04)	<.001	0.03 (0.02-0.04)	<.001	0.03 (0.02-0.04)	<.001	n.a.	n.a.
**Problem Drinking**										
Workplace incivility	2.66 (1.01-7.04)	0.048	2.79 (1.02-7.62)	0.045					2.24 (0.78-6.42)	0.131
Identity-based harassment	1.15 (0.56-2.34)	0.704			1.31 (0.60-2.85)	0.491			0.97 (0.42-2.21)	0.934
Threats and violence	2.39 (1.23-4.64)	0.010					2.38 (1.20-4.73)	0.013	2.19 (1.08-4.47)	0.030
Male (ref female)			1.81 (0.94-3.50)	0.075	1.91 (0.97-3.78)	0.062	1.85 (0.96-3.56)	0.068	1.82 (0.92-3.63)	0.087

Rank					.			.
Junior and intern physician	2.29 (0.82-6.35)	0.112	2.29 (0.82-6.40)	0.114	2.01 (0.72-5.61)	0.185	1.97 (0.69-5.60)	0.202
Resident	1.04 (0.38-2.82)	0.937	1.03 (0.38-2.82)	0.950	0.95 (0.35-2.57)	0.919	0.93 (0.34-2.56)	0.887
Specialist	1.48 (0.55-3.97)	0.435	1.48 (0.55-3.96)	0.435	1.41 (0.52-3.78)	0.499	1.41 (0.52-3.82)	0.493
Consultant	.	.	.	.	.	.	.	.
Workplace								
Primary Care	0.73 (0.21-2.49)	0.617	0.65 (0.19-2.19)	0.491	0.72 (0.21-2.44)	0.599	0.79 (0.23-2.71)	0.706
Hospital	0.98 (0.32-3.00)	0.973	0.89 (0.30-2.71)	0.846	0.98 (0.32-3.01)	0.979	1.05 (0.34-3.22)	0.934
Other	.	.	.	.	.	.	.	.
Country of birth								
Sweden	0.59 (0.13-2.59)	0.481	0.56 (0.13-2.48)	0.446	0.52 (0.32-3.01)	0.388	0.54 (0.12-2.42)	0.421
Inside Europe[f]	.	.	.	.	.	.	.	.
Outside Europe	.	.	.	.	.	.	.	.
Baseline Alcohol consumption[g]	11965320.282 (same)	<.001	12049508.233 (same)	<.001	11474995.204 (same)	<.001	11559419.637 (same)	<.001

[a] Univariate mode
[b] Model 2a: -2 log likelihood=333.474 (P<0.001).
[c] Model 2b: -2 log likelihood=375.733(P<0.001).
[d] Model 2c. -2 log likelihood=385.280 (P<0.001).
[e] Model 3. -2 log likelihood=534.513 (P<0.001).
[f] Within Europe except Sweden;
[g] Baseline alcohol consumption was entered as a categorical covariate with three levels: abstaining, moderate drinking, and problem drinking.

Table S3. Fully adjusted Model 3 stratified by women and men

	Women		Men	
	**OR (95% CI)**	**P-value**	**OR (95% CI)**	**P-value**
**Abstaining**				
Workplace incivility	1.66 (0.66-4.17)	0.278	2.44 (0.87-6.84)	0.090
Identity-based harassment	0.89 (0.52-1.53)	0.678	1.91 (0.81-4.51)	0.141
Threats and violence	0.79 (0.43-1.43)	0.785	1.00 (0.46-2.17)	0.993
**Problem Drinking**				
Workplace incivility	2.51 (0.64-9.80)	0.186	2.05 (0.41-10.20)	0.380
Identity-based harassment	1.21 (0.45-3.27)	0.702	0.56 (0.14-2.19)	0.403
Threats and violence	3.30 (1.25-8.76)	0.016	2.13 (0.80-5.65)	0.129

Model adjusted by rank, workplace country of birth and baseline alcohol consumption
